# A Calibrated Modelling Approach for Predicting Dry Friction Wear of Copper-Free Composite Friction Materials

**DOI:** 10.3390/ma19132831

**Published:** 2026-07-02

**Authors:** Grzegorz Mieczkowski, Andrzej Borawski, Dariusz Szpica

**Affiliations:** Faculty of Mechanical Engineering, Bialystok University of Technology, 45A Wiejska Str., 15-351 Bialystok, Poland; g.mieczkowski@pb.edu.pl (G.M.); a.borawski@pb.edu.pl (A.B.)

**Keywords:** friction materials, copper-free composites, brake pads, dry wear, predictive modelling

## Abstract

**Highlights:**

- Copper was replaced by Al/PTFE in composite brake friction materials.
- Ball-cratering tests quantified dry abrasive wear in four material variants.
- Four calibrated predictive models were compared using wear-rate data.
- The Hertz–Archard-based model achieved the best prediction accuracy.
- The approach supports copper-free friction material design with controlled wear.
- The approach supports screening of copper-free friction-material formulations.

**Abstract:**

This study presents a calibrated modelling approach for predicting the abrasive wear of copper-free composite friction materials. Four formulations were analysed, including a copper-containing reference material and three experimental compositions in which copper was replaced by different aluminium/polytetrafluoroethylene ratios. Dry ball-cratering tests were performed to determine the apparent wear-rate coefficient under controlled laboratory conditions. The copper-containing reference material showed the lowest wear-rate coefficient, *k_c_* = 80.655 × 10^−14^ m^2^·N^−1^, whereas the copper-free formulations reached *k_c_* = 111.811 × 10^−14^ m^2^·N^−1^, 98.586 × 10^−14^ m^2^·N^−1^ and 90.579 × 10^−14^ m^2^·N^−1^ for S2, S3 and S4, respectively. Thus, copper replacement increased the apparent wear-rate coefficient by approximately 12–39%, depending on the Al/PTFE ratio. The obtained data were used to develop and compare four calibrated predictive models. Among them, the modified Hertz–Archard model, which included effective hardness and contact-related descriptors, provided the best agreement with the experimental data. This model achieved MAPE = 1.5%, RMSE = 2.181 × 10^−14^ m^2^·N^−1^ and a maximum absolute error of 4.3%, with all predictions within the ±5% error band. The results indicate that the proposed calibration framework can support preliminary screening and ranking of copper-free friction-material formulations under the adopted dry ball-cratering conditions.

## 1. Introduction

Composite materials are currently one of the most important groups of engineering materials because they allow functional properties to be designed through the deliberate selection of the matrix, reinforcing phases and functional additives. Their advantage over traditional homogeneous materials results primarily from the possibility of combining properties that are difficult to achieve simultaneously in a single material, such as adequate strength, wear resistance, thermal stability, vibration damping, low mass and environmental resistance [1,2,3]. In components operating under frictional contact, it is particularly important to control not only mechanical properties, but also the tribological response, including the coefficient of friction, wear rate, fade resistance, ability to form a transfer layer and stability under variable load and temperature conditions [2,3,4,5,6,7].

In the automotive industry, composites are widely used in both structural components and functional systems. In lightweight vehicle structures, they are used mainly to reduce mass while maintaining adequate stiffness and strength [1,8]. A particularly important application group is represented by composite friction materials, which are used in brake pads, brake linings, clutches and other components that transmit loads through frictional contact [1,3,9,10]. In these applications, the material cannot be evaluated solely based on static strength. It must provide stable friction, controlled wear, resistance to high temperature, adequate heat conduction, limited emission of wear particles and an acceptable level of noise and vibration [4,5,6,7,11]. For this reason, friction materials are typical multi-component composites in which each constituent performs a specific function.

Modern brake pads are usually composite materials containing a binder, reinforcing fibres, fillers, and wear and friction modifiers [2,3,12]. The binder is most often a phenolic resin or another thermosetting resin, which ensures the integrity of the material and holds the constituents within one structure. Fibres, such as aramid, steel, carbon, mineral or natural fibres, are responsible for mechanical reinforcement, crack resistance and dimensional stability [13,14,15]. Fillers, including barite, fly ash, minerals or industrial wastes, are used to control density, thermal conductivity, cost and processing stability [2,16]. Abrasive constituents, such as metal oxides, ceramics, steel or cast iron, increase friction efficiency and the ability to clean the counter surface, but they may also intensify wear [7,11,17,18]. In contrast, lubricating or film-forming constituents, such as graphite, copper, sulphides, PTFE and other additives with low shear strength, stabilise friction, reduce adhesion and promote the formation of a transfer layer [7,19].

From the point of view of brake-system operation, the formation of the so-called third body and tribological layers at the pad–disc interface is particularly important. Studies of the surfaces of organic friction materials have shown that real contact does not occur over the entire nominal area, but through local primary and secondary plateaus formed by compaction and transformation of wear products [4,5,6,11]. The nature of these layers depends on material composition, temperature, pressure, sliding velocity and running-in history. Therefore, the effectiveness of a given additive does not result solely from its intrinsic material properties, but also from its contribution to the formation and stabilisation of the transfer layer. For this reason, the design of friction materials is a multi-criteria task in which mechanical, thermal, chemical and tribological properties must be considered simultaneously [2,4,6,11].

Copper has historically been one of the most important additives used in friction materials. Its presence improves the thermal conductivity of the material, stabilises the coefficient of friction, reduces local overheating, decreases the tendency to generate noise and promotes the formation of beneficial layers on the friction surface [7,19]. Österle et al. showed that the role of copper is not limited to heat conduction, but also includes participation in tribolayer formation and contact stabilisation [6]. Therefore, replacing copper with a single constituent is difficult because its thermal, lubricating and stabilising functions must be reproduced simultaneously. At the same time, the emission of copper-containing wear particles has become an important environmental issue. Particles generated by brake-pad wear can enter air, soil and water, and their chemical composition, size and toxicity have been the subject of numerous studies [17,18,20,21]. For this reason, the development of copper-free or low-copper friction materials is currently one of the most important research directions in braking systems with reduced copper emissions. The significance of copper in brake pads and the need to develop substitutes have been confirmed by studies on Cu-free brake pads and particle emissions from copper-free materials.

Copper performs several beneficial functions in brake friction materials. It improves thermal conductivity, contributes to contact stabilisation and may act as a solid-lubricant-type constituent. Therefore, replacing copper requires a functional combination of additives rather than a simple one-to-one substitution. In the present study, aluminium and polytetrafluoroethylene (PTFE) were selected as a model replacement system intended to reproduce, at least partially, the two main functions of copper. PTFE was introduced mainly as a low-shear and anti-adhesive constituent capable of supporting the formation of a lubricating transfer layer, whereas aluminium was introduced as a thermally conductive and relatively stiff metallic constituent. Thus, the expected effect of the Al/PTFE system is based on a functional division of copper’s role: PTFE contributes primarily to solid-lubrication behaviour, while aluminium contributes mainly to heat dissipation and contact stabilisation. The selection of the Al/PTFE system was additionally motivated by our previous experimental study, which demonstrated that aluminium and PTFE may partially reproduce selected tribological functions traditionally associated with copper in friction materials. Therefore, the present work focuses on this particular copper-free system as a representative case study for validating the proposed wear-prediction methodology.

It should be noted that replacing copper with PTFE-containing systems does not automatically eliminate all environmental concerns associated with brake friction materials. PTFE is a fluoropolymer and is therefore discussed in the broader context of PFAS-related regulatory and environmental debates [22,23]. For this reason, the Al/PTFE system analysed in this work should be treated as a model copper-free formulation used to evaluate the proposed wear-prediction methodology, rather than as a final environmentally neutral replacement for copper. A full assessment of its suitability would require additional studies on particle emissions, possible thermal degradation products and life-cycle aspects.

Research on copper-free friction composites is being conducted in several main directions. The first involves replacing copper with other metallic or conductive constituents, such as stainless steel, steel swarf, stainless-steel particles, metallic fibres, graphite or mineral materials [19,20,21,24,25,26]. Aranganathan and Bijwe proposed environmentally friendly copper-free friction materials based on new functional ingredients and showed that proper additive selection can provide stable tribological properties [24]. Mahale, Bijwe and Sinha investigated the possibility of replacing copper with stainless-steel swarf, indicating the potential of this additive as a substitute for the metallic constituent [25]. Subsequent studies also analysed the effect of plasma treatment of stainless steel on the wear resistance of copper-free materials [26,27]. Kalel et al. demonstrated that the appropriate selection of the type and morphology of stainless-steel particles can be crucial for obtaining acceptable friction and wear properties in Cu-free materials [28].

The second research direction concerns the use of natural, waste-derived or plant-origin additives as reinforcing constituents or fillers. The literature describes friction materials containing hemp fibres, flax fibres, organic fibres, fly ash and plant wastes [13,14,15,16,29,30]. These studies result not only from the need to eliminate copper, but also from the broader trend of reducing toxic or energy-intensive constituents in friction materials. Naidu et al. investigated brake composites reinforced with hemp fibres and indicated the potential of natural fibres for reducing the environmental impact of friction materials [29]. Borawski et al. showed that flax fibres can be considered as an alternative to conventional aramid fibres in composite friction materials [13]. In turn, Mohanty and Chugh, as well as Idris et al., analysed the possibility of using fly ash and plant wastes to develop more environmentally friendly friction materials [9,16]. These studies show that the development of friction materials increasingly combines tribological requirements with environmental and economic aspects.

Borawski et al. have also made an important contribution to research on composite friction materials. In a review paper on composites in vehicle braking systems, the authors identified key development areas, including composition design, tribological testing, emission of wear products and modelling of phenomena occurring in braking systems [1]. Other studies analysed the effect of brake-pad service time on tribological properties [31], compared results obtained using pin-on-disc and ball-cratering methods [32], investigated the effect of the shape of the copper constituent on friction-material properties [33] and assessed the possibility of replacing copper with a PTFE/aluminium system [14]. The latter work is directly related to the subject of the present article, as it concerns the use of aluminium and PTFE as functional substitutes for copper in composite friction materials. It was shown that the Al/PTFE system affects the coefficient of friction and wear rate, which justifies further research aimed at quantitatively describing the relationship between composition and wear resistance [14]. These sources confirm the relevance of composition design and test-method validation for friction materials used in brakes.

Most studies on composite friction materials are experimental in nature. Pin-on-disc, ball-cratering, brake-test rigs and dynamometers are commonly used, followed by analysis of the coefficient of friction, wear intensity, temperature, surface morphology and composition of wear products [9,11,15,16,17,18,20,21,29,30,31,32]. Experimental testing is indispensable because it enables verification of the real behaviour of the material under frictional contact. However, its limitations include cost, time consumption and the need to perform many test series for each change in composition. In multi-component materials, the number of possible additive combinations is very large; therefore, experimental testing alone is not sufficient for the efficient design of new formulations. Models are needed to narrow the search space, identify the most promising compositions and better understand the influence of particular groups of constituents on wear.

In the literature, friction materials have been modelled using various approaches. The most classical basis for describing wear is Archard’s law, in which the wear volume is related to the normal load, sliding distance and wear resistance of the material [34]. In contact analyses, Hertz theory and classical contact mechanics are also frequently used to estimate the size of the contact zone, contact pressure and the effect of the elastic properties of the materials [35,36]. More advanced approaches include finite element models, coupled thermo-mechanical models, models with updating of the worn surface geometry, regression models and models based on test-rig data [34,37,38,39,40,41]. AbuBakar and Ouyang applied the finite element method to predict friction-material wear and analyse brake squeal [42]. Hatam and Khalkhali performed simulations and sensitivity analyses of brake-pad wear for selected parameters [35]. Sawczuk et al. proposed a model of mass wear of railway disc-brake friction pads based on test-rig data [36], whereas Grześ and Kuciej developed a finite element model coupling thermal, structural and wear phenomena in a braking system [39]. Such approaches are useful because they allow the analysis of pressure, temperature, stress and wear-depth distributions that are difficult or impossible to measure directly.

Despite the development of numerical methods, a gap still exists between detailed modelling of the braking system and practical design of the friction-material composition. Finite element models usually require extensive input data, system geometry, boundary conditions and substantial computational effort. Regression models, in turn, often describe a specific dataset well, but their physical interpretability is limited. For friction materials, an intermediate approach is therefore particularly valuable, combining the simplicity of empirical models with selected material descriptors, such as the fraction of hard phases, the fraction of film-forming phases, effective hardness, elastic properties and contact parameters. This makes it possible not only to fit the model to experimental data, but also to interpret which compositional features are responsible for changes in wear rate.

The novelty of the present work lies in combining original ball-cratering wear-test results with the development and comparison of several calibrated predictive models based on material composition and effective material descriptors. In contrast to purely experimental studies, in which only prepared formulations are evaluated, the present work proposes a procedure for predicting the wear-rate coefficient from the fractions of constituents. At the same time, unlike advanced finite element models, the aim was not to reproduce the full geometry of the braking system, but to create a practical tool supporting friction-material formulation design. Particular importance is attached to the comparison of four models with different levels of complexity: a hard-phase/tribological-film model, a logarithmic model with an Al/PTFE interaction term, an Archard-type model extended by effective hardness and Hertzian pressure, and a simplified linear composition-based model. This comparison makes it possible to assess whether increased physical interpretability and the introduction of contact parameters improve wear-prediction accuracy.

This article presents a study of composite friction materials in which a conventional copper-containing formulation was compared with three copper-free compositions based on different aluminium/PTFE ratios. Section 2 describes the composition and preparation of the materials, the ball-cratering test procedure and the experimental dry wear results. The following section presents the modelling assumptions, the description of four predictive models, the calibration procedure and the comparison of prediction accuracy. Finally, the modelling results are compared with the experimental data in order to identify the model that best describes the effect of compositional changes on abrasive wear rate. This approach aims not only to evaluate a specific Al/PTFE system as a substitute for copper, but also to propose a methodology supporting the design of new copper-free composite friction materials.

## 2. Experimental Procedure and Wear Test Results

### 2.1. Composition and Preparation of Friction Materials

The investigated materials were multi-component friction composites intended for brake-system applications. Four formulations, denoted as S1–S4, were prepared, with three specimens produced for each composition. Specimen S1 was used as the copper-containing reference material, whereas in specimens S2–S4 copper was completely removed and replaced by different aluminium/PTFE ratios. This formulation strategy enabled the effect of copper substitution and the relative proportion of aluminium and PTFE on abrasive wear behaviour to be analysed.

PTFE powder with particle sizes of 20–70 µm and aluminium powder with an average particle size of approximately 65 µm were used to prepare the copper-free formulations. The remaining constituents, including steel, aramid fibres, resin, fly ash and cast iron, were kept constant in all specimens. The detailed compositions of the prepared materials are presented in Table 1.

As shown in Table 1, the copper-containing reference formulation S1 contained 20 wt.% Cu. In specimens S2–S4, copper was fully eliminated and replaced by aluminium and PTFE in different proportions, while the contents of the other constituents remained unchanged. This design allowed the influence of the Al/PTFE ratio on the wear response of the copper-free materials to be isolated.

**Figure 1:**
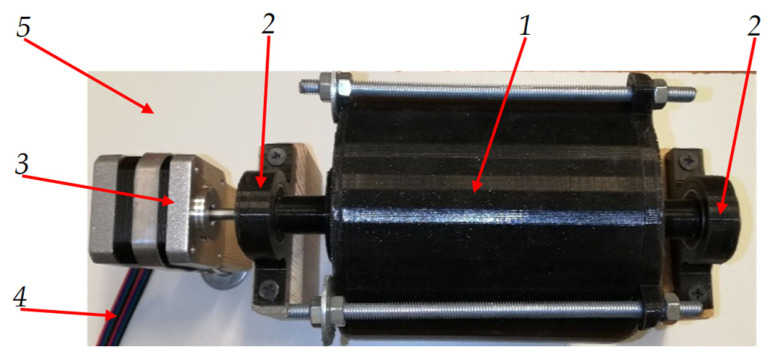
Component mixing device: 1—mixing tube, 2—bearing, 3—stepper motor, 4—connector, 5—base [14].

In each case, the appropriate amounts of constituents were weighed using a Steinberg SBS-LW-300A precision balance with an accuracy of 10^−3^ g (Hamburg, Germany). The weighed constituents were then placed in a mixing device manufactured by additive technology, shown in Figure 1. Mixing was carried out for 1 h at a rotational speed of 50 r./min. The internal mixing ribs ensured homogenisation of the composite constituents and reduced the risk of local powder segregation before the subsequent specimen-forming stage.

The prepared mixture was placed in cylindrical metal moulds with an internal diameter of 1 inch, i.e., 25.4 mm. The material was then compacted by applying a pressure of 20 MPa to the uncured specimen. The formed specimens were heat-treated for 24 h at 55–60 °C in order to consolidate the composite structure and ensure adequate material stabilisation. After heat treatment, the front surfaces of the specimens were ground. The aim of this stage was to obtain the required flatness tolerance and a surface roughness on the order of 10^−6^ m, ensuring repeatable contact conditions during tribological testing.

### 2.2. Ball-Cratering Test Procedure

**Figure 2:**
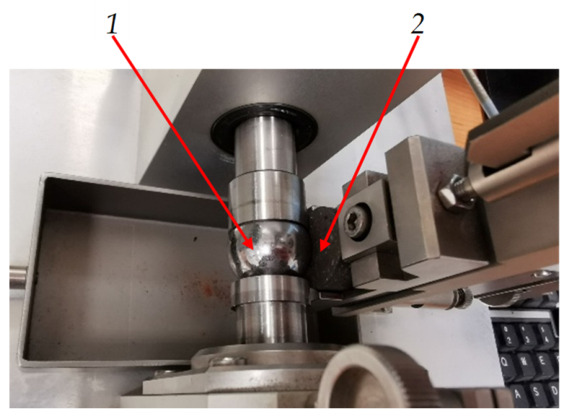
T-20 test stand: 1—sample, 2—counter-sample.

The tests were performed using a T-20 laboratory test rig (Instytut Technologii Eksploatacji–Państwowy Instytut Badawczy, Radom, Poland) operating in a ball-on-disc configuration (Figure 2). The T-20 tribometer operates in a ball-cratering configuration, in which a rotating steel ball is pressed against the flat surface of the tested specimen under a controlled normal load. During the test, the ball performs rotational motion while maintaining continuous contact with the specimen, resulting in the formation of a wear crater. The dimensions of the crater are subsequently used to determine the wear-rate coefficient. Although the adopted configuration does not reproduce all physical phenomena occurring in an actual braking system, it provides a repeatable and widely accepted laboratory method for comparative evaluation of the abrasive wear resistance of friction materials under controlled conditions.

The tests were conducted under dry ball-cratering conditions, without any externally supplied abrasive slurry or loose abrasive particles. Consequently, no external abrasive material, particle-size distribution or slurry concentration is reported for the present tests. This distinction is important because the adopted procedure should not be interpreted as a classical slurry-based micro-scale abrasion test. In this work, the wear-rate coefficient describes the material removal generated in dry ball–specimen contact, where the wear process is controlled by the tested composite, the steel counter-sample ball, hard constituents present in the friction material and self-generated wear debris. This rig has been used many times for this type of testing, and the detailed methodology has been described in [43].

In the ball-cratering method, proper experimental preparation and selection of test parameters are of particular importance. In the literature, this stage is carried out in various ways; however, one of the most commonly used and effective methods for planning multi-parameter experiments is the Taguchi method [44,45]. It enables rational selection of input-parameter levels in order to reduce the number of tests while maintaining high reliability of the results and minimising the relative measurement error.

In classical micro-scale abrasion tests with an externally supplied slurry, the dominant wear mode may change between grooving/sliding abrasion, rolling abrasion and mixed abrasion, and such transitions are strongly affected by normal load and abrasive-particle concentration [46,47]. Since no abrasive slurry was used in the present study, the slurry-concentration-based wear-mode maps cannot be applied directly. The observed crater morphology was therefore used only as qualitative evidence for identifying the dominant dry-contact wear features under the adopted test conditions.

The first stage of the procedure is the preparation or selection of an appropriate orthogonal array. In the analysed case, three basic input parameters were adopted: ball rotational speed *n*, sliding distance *S* and normal load *L*. For a system involving three independent variables, an orthogonal array consisting of nine experimental variants is applicable [48]. The array used to conduct the preliminary tests, developed on the basis of the standard [49], is presented in Table 2.

The preliminary tests were performed using a specimen with composition S1. To minimise the risk of measurement error, each test was repeated five times. The results obtained in these tests are presented in Table 3.

The results of the preliminary tests were analysed using the Taguchi signal-to-noise ratio with the “smaller-is-better” criterion, described by the relationship:

(1) $$\eta =-10\;\log_{10}\left(\frac{1}{m}\sum_{i=1}^{m}x_{i}^{2}\right)$$

where *m* is the number of repeated measurements performed for a given set of test parameters and xi is the crater diameter measured in the *i*-th repetition. The value of *η* is a statistical quality index used to compare different combinations of test parameters in terms of response level and repeatability. Under the adopted “smaller-is-better” criterion, a higher value of *η* indicates more favourable test conditions, i.e., measurable and repeatable craters with limited material removal. Based on this analysis and by using S1 samples, the following parameters were selected for the main tests:

- Normal load: 2 N;
- Sliding distance: 50 m;
- Ball rotational speed: 38 r./min.

The sliding distance was variable only in the preliminary parameter-selection matrix in order to obtain measurable and reproducible craters. The final comparative tests and the subsequent modelling were performed at a fixed sliding distance of *S* = 50 m. Because a separate wear-volume-versus-distance study was not performed, the calculated coefficient should be interpreted as an apparent dry ball-cratering wear-rate coefficient determined under the adopted test conditions, rather than as a fully general steady-state wear coefficient for arbitrary sliding distances.

The next stage was to determine the abrasive wear-rate coefficient of the prepared materials. This coefficient was determined from the dimensions of the craters formed on the specimen surfaces during the ball-cratering test. The crater diameter was measured in two mutually perpendicular directions: along the friction direction (*b*_1_) and perpendicular to this direction (*b*_2_). A Delta Optical microscope and a Brinell magnifier were used for the measurements. The wear craters were not always perfectly circular. Slight deviations from the ideal crater geometry were attributed to anisotropic wear resulting from the sliding motion, heterogeneous distribution of the composite constituents and local interactions between the steel ball and hard reinforcement particles. Therefore, the crater diameter was measured in two mutually perpendicular directions and the arithmetic mean was used in subsequent calculations. This approach minimises the effect of local shape irregularities and is commonly applied in ball-cratering wear measurements to improve the accuracy of crater-size determination. The final crater diameter was determined as the arithmetic mean of both measurements:

(2) $$\eta =-10\;\log_{10}\left(\frac{1}{m}\sum_{i=1}^{m}x_{i}^{2}\right)$$

The determined crater diameter was then substituted into the relationship derived from Archard’s law, which, after transformation for the spherical crater geometry, takes the form [49,50]:

(3) $$k_{c}=\frac{\pi b^{4}}{64RLS}$$

where *R* is the radius of the steel counter-sample ball. The ball diameter was 12.7 mm, corresponding to *R* = 6.35 mm.

The basic form of Archard’s law can be written as:

(4) $$V=k_{c}SL$$

where *V* is the volume of material removed from the specimen during the test.

This relationship allows both the volume and, after accounting for material density, the mass of wear products to be determined.

**Figure 3:**
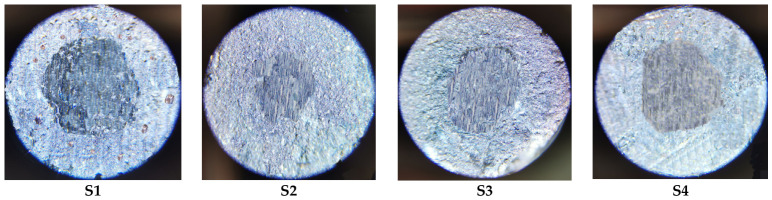
Representative optical images of wear craters obtained in the dry ball-cratering test for specimens S1–S4. The images are used as qualitative support for the identification of the dominant sliding/grooving-type dry wear features; the volume fractions used in the models were calculated from mass fractions and density data, not from image analysis.

Representative optical images of the wear craters obtained for specimens S1–S4 are presented in Figure 3. The crater interiors show predominantly directional grooves and local ploughing/smearing features aligned with the sliding direction. This morphology is consistent with a sliding/grooving-dominated dry-contact wear mechanism, with a possible contribution of self-generated third-body debris. Because no external abrasive particles were supplied, the observed wear mode should not be interpreted as slurry-mediated rolling abrasion.

### 2.3. Experimental Abrasive Wear Results

One of the direct results of the ball-cratering test was the formation of characteristic depressions on the specimen surfaces, referred to as wear craters. Measuring their dimensions made it possible to determine the abrasive wear-rate coefficient *k_c_* for each material group. For this purpose, the crater diameters determined according to Equation (2) were substituted into the relationship described by Equation (3). On this basis, *k_c_* values were calculated for each individual test. Accordingly, the calculated values of *k_c_* should be interpreted as apparent dry ball-cratering wear-rate coefficients corresponding to the adopted test conditions and should not be treated as universal material constants independent of loading conditions, contact geometry, counter-sample material, temperature or sliding distance.

It should be emphasised that the optical images were not used for quantitative phase-volume analysis. They were included to document the crater morphology and to support the qualitative interpretation of the wear mode. The volume fractions used in the predictive models were nominal values calculated from the weighed mass fractions and constituent densities, as described in Appendix A.

In the next stage, the mean values and standard deviations were determined for each specimen group using the same statistical procedure for all wear-rate results. This made it possible to assess both the wear level of individual materials and the repeatability of the experimental results. The calculated values of the abrasive wear-rate coefficient are summarised in Table 4.

The mean values of *k_c_* calculated for each material group were used as the experimental reference values in the subsequent model calibration and comparison. The individual repeated measurements were used to determine the standard deviation and coefficient of variation (CV), which confirmed good experimental repeatability. The calculated CV values were 2.76% for S1, 2.50% for S2, 2.13% for S3 and 2.41% for S4. Because the scatter levels were low and comparable between the investigated materials, equal weights were used in the final calibration. However, the general calibration formulation also allows standard-deviation-based weighting, as described in Appendix A.

For material S1, which served as the reference specimen, the wear-rate coefficient *k_c_* was 80.655 × 10^−14^ m^2^·N^−1^. Complete removal of copper from the material composition and its replacement with the aluminium/PTFE system increased this coefficient in all analysed copper-free compositions. The smallest increase, approximately 15%, was recorded for material S4, which had the lowest PTFE content and the highest aluminium fraction. The largest increase in *k_c_*, reaching approximately 25%, was obtained for specimen S2, which had the highest PTFE content and the lowest aluminium fraction.

The obtained results indicate that the presence of a constituent with lubricating properties alone does not fully reproduce the function of copper in a friction material. The mutual proportion of the constituents in the Al/PTFE system is also important, since increasing the aluminium fraction while reducing the PTFE content led to a decrease in the abrasive wear rate.

Based on the determined *k_c_* values and Equation (4), the volumetric material loss per metre of sliding distance was calculated for the individual specimen groups. The results of these calculations are presented in Table 5.

## 3. Predictive Wear Modelling and Model Calibration

The experimental results presented in the previous section were used as the basis for developing and evaluating predictive models of abrasive wear. The purpose of this stage was not only to reproduce the measured values of the wear-rate coefficient, but also to assess whether the effect of compositional changes, especially copper replacement by the Al/PTFE system, can be described using relatively simple relationships based on composition and material properties. For this reason, four model variants were formulated and compared. Each model used the material composition and selected effective properties as input variables, whereas the experimentally determined wear-rate coefficient was used as the reference output for calibration and validation.

The modelling procedure consisted of four main steps: conversion of mass fractions into volume fractions, calculation of effective material descriptors, determination of the raw model response and calibration of the predicted wear-rate coefficient against selected experimental data. The calibrated models were then evaluated by comparing predicted and experimental values using standard error measures.

### 3.1. General Modelling Approach

The modelling stage was designed as a calibrated screening procedure for comparing the response of the investigated copper-containing and copper-free friction materials. The abrasive wear-rate coefficient determined from the ball-cratering tests was used as the reference output. In the present modelling framework, this coefficient was used only as an apparent experimental descriptor for calibration and comparison within the investigated material system and test configuration. Material composition, phase grouping and selected effective descriptors were used as input variables. The density, hardness and elastic-property values used for the mass-to-volume conversion and Hertzian contact calculations are summarised in Table A1. Detailed equations for converting mass fractions into volume fractions, calculating effective hardness and determining the raw model response are provided in Appendix A.

### 3.2. Overview of Predictive Models

Four model variants were considered. They were not intended to replace classical wear theories, but to provide semi-empirical predictive formulations suitable for ranking and comparing model structures within the investigated composition range. The complete mathematical formulation of the models is given in Appendix A, whereas their conceptual assumptions are summarised in Table 6.

For this reason, the proposed M0–M3 relationships should be understood as calibrated semi-empirical formulations developed for comparative screening of the analysed friction-material compositions. They are not universal wear laws and should not be extrapolated beyond the investigated composition range, loading condition and identified wear mode without additional validation.

Model M0 represents the competition between abrasive hard phases and film-forming constituents. Model M1 describes the interaction between aluminium and PTFE using a logarithmic empirical formulation. Model M2 is a modified Hertz–Archard model that combines an Archard-type wear description with effective hardness and Hertzian contact descriptors. Model M3 is a simplified linear composition-based model introduced as a reference variant.

### 3.3. Model Calibration Procedure

Each model first produced an uncalibrated value of the wear-rate coefficient. This raw response was then corrected using the same scaling calibration coefficient. In the final comparison, specimens S1 and S4 were used as calibration points because they represented the copper-containing reference material and the copper-free formulation with the highest aluminium and lowest PTFE content, respectively. Specimens S2 and S3 were not used to determine the calibration coefficient and were treated as validation points for assessing whether the calibrated models could reproduce the trend caused by changes in the Al/PTFE ratio. The analytical expressions for the calibration coefficient and the optional weighting procedure are presented in Appendix A.

### 3.4. Model Parameters Adopted in the Calculations

The structural and empirical parameters used in the predictive models were treated as model-shape parameters rather than universal material constants. Their role was to define the relative sensitivity of each formulation to hard/abrasive phases, film-forming constituents, effective hardness (*H_eff_*) and the Al/PTFE interaction. The absolute level of the predicted wear-rate coefficient was adjusted separately by the calibration coefficient C, determined from the selected calibration specimens.

This approach was adopted to reduce the risk of overfitting to the limited number of tested material formulations and to preserve the comparability of the analysed model variants. The complete list of adopted parameters, their values and the physical interpretation is provided in Appendix A, Table A2.

It should also be noted that *H_eff_* used in the models was not measured directly as the hardness of the whole composite. It was calculated from constituent properties using the mixture rules described in Appendix A.2 and should therefore be interpreted as an equivalent model descriptor. The detailed justification for this approach, including the selected harmonic mixing rule, is given in Appendix A.

### 3.5. Prediction Accuracy and Model Comparison

The predictive capability of the four proposed models was evaluated by comparing the predicted values of the abrasive wear-rate coefficient with the corresponding experimental values obtained for specimens S1–S4. The comparison was performed using standard error measures, i.e., mean absolute percentage error (MAPE), root mean square error (RMSE), mean bias and maximum absolute percentage error. These measures were used both to assess the average accuracy of the models and to evaluate prediction stability for individual specimens.

Four error measures were used for the quantitative assessment of prediction accuracy. For each specimen, the relative error between the predicted value *k_c_*_,pred,*i*_ and the experimental value *k_c_*_,exp,*i*_ was calculated. The mean absolute percentage error, MAPE, describes the average value of the relative error regardless of its sign:

(5) $$\mathrm{MAPE}=\frac{100\%}{n}\sum_{i=1}^{n}\left|\frac{k_{c,\mathrm{pred},i}-k_{c,\exp,i}}{k_{c,\exp,i}}\right|.$$

The root mean square error, RMSE, defines the absolute scale of deviations in the units of the wear-rate coefficient:

(6) $$\mathrm{RMSE}=\sqrt{\frac{1}{n}\sum_{i=1}^{n}{\left(k_{c,\mathrm{pred},i}-k_{c,\exp,i}\right)}^{2}}.$$

The mean bias was calculated as the average difference between the predicted and experimental values:

(7) $$\mathrm{Bias}=\frac{1}{n}\sum_{i=1}^{n}\left(k_{c,\mathrm{pred},i}-k_{c,\exp,i}\right).$$

In addition, the maximum absolute percentage error was determined in order to assess the largest local deviation of the model from the experimental result:

(8) $$E_{\max}=100\%\max_{i}\left|\frac{k_{c,\mathrm{pred},i}-k_{c,\exp,i}}{k_{c,\exp,i}}\right|,$$

where *n* denotes the number of specimens used to evaluate model accuracy. In the present case, *n* = 4 because the comparison was performed for specimens S1–S4. The calibration coefficient was determined from S1 and S4, whereas S2 and S3 were used as validation points for assessing the ability of the models to reproduce the compositional trend within the investigated Al/PTFE range.

The values of the main accuracy measures are summarised in Table 7. Among the analysed variants, Model M2 showed the best agreement with the experimental data. It achieved the lowest values of MAPE (1.5%), RMSE (2.181 × 10^−14^ m^2^·N^−1^) and maximum absolute error (4.3%). Moreover, all predictions of Model M2 were within the ±5% error band. This result suggests that the inclusion of effective hardness and contact-related descriptors may contribute to improved predictive capability. However, because the analysed models differ simultaneously in their mathematical structure, constitutive descriptors and adopted parameters, the present dataset does not allow the individual contribution of each modelling assumption to be isolated. In contrast, Model M3 had the weakest overall accuracy, with the highest MAPE (7.4%), RMSE (11.496 × 10^−14^ m^2^·N^−1^) and the largest maximum error (19.2%). The lowest absolute bias was obtained for Model M0, indicating the smallest systematic deviation between prediction and experiment.

A more detailed comparison of errors for individual specimens is presented in Table 8. The percentage prediction errors show that the models differed not only in their average accuracy, but also in their behaviour for specific material compositions. Model M2 was the most stable variant because it provided the most accurate prediction for specimens S1, S2 and S4, and its error remained within ±5% for all specimens. Model M0 gave the best result only for specimen S3, but exceeded the ±5% band for specimens S1 and S4. Model M1 showed intermediate accuracy, with three predictions within the ±5% limit, whereas Model M3 generated the largest deviations, particularly for specimen S2, for which the error was −19.2%.

The overall ability of the models to satisfy the adopted ±5% acceptance criterion is summarised in Table 9. Model M2 met this condition for all four analysed specimens, whereas Model M1 did so for three specimens. Models M0 and M3 were within the ±5% band for only two specimens. For Model M0, exceedances occurred for specimens S1 and S4, whereas for Model M3 they occurred for S2 and S3. This comparison confirms that Model M2 exhibited the highest prediction stability for the individual material compositions.

The supplementary ranking comparison is presented in Table 10.

It includes the best and second-best models according to selected accuracy criteria, i.e., MAPE, RMSE, absolute bias and maximum absolute error. Model M2 achieved the best result for three of the four criteria: MAPE, RMSE and maximum error. The lowest absolute bias was obtained for Model M0, which indicates the smallest average prediction shift relative to the experimental data. However, considering all criteria jointly, Model M2 can be regarded as the variant with the best overall accuracy.

**Figure 4:**
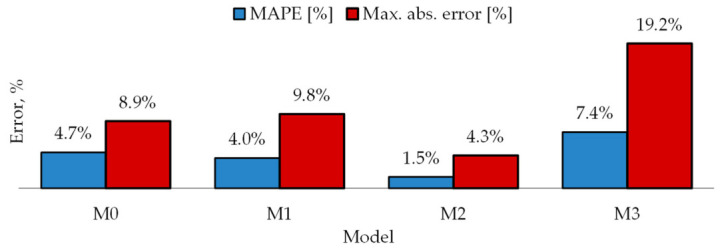
Comparison of model accuracy metrics: MAPE and maximum absolute percentage error.

A graphical comparison of the main error measures is shown in Figure 4. The chart shows that Model M2 was characterised by the lowest MAPE and the lowest maximum absolute error. The highest values of both measures were obtained for Model M3, confirming that the simplified linear description of composition was insufficient for accurately reproducing the experimental results.

**Figure 5:**
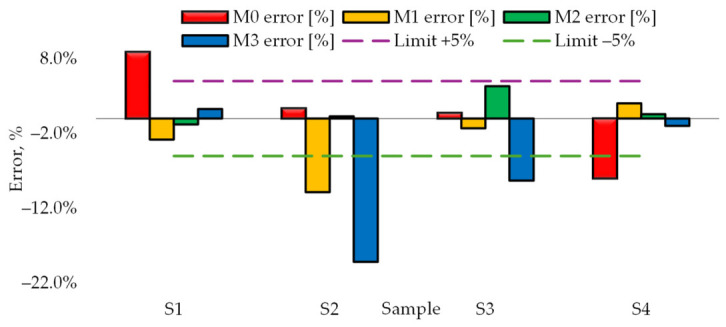
Signed percentage prediction error for each sample and model. The dashed horizontal lines indicate the ±5% limits.

The distribution of percentage errors for individual specimens is presented in Figure 5. This chart makes it possible to identify which material compositions were overestimated or underestimated by the individual models. Model M2 remained within the ±5% limits for all specimens, whereas the largest local deviation was obtained for Model M3 in the case of specimen S2.

**Figure 6:**
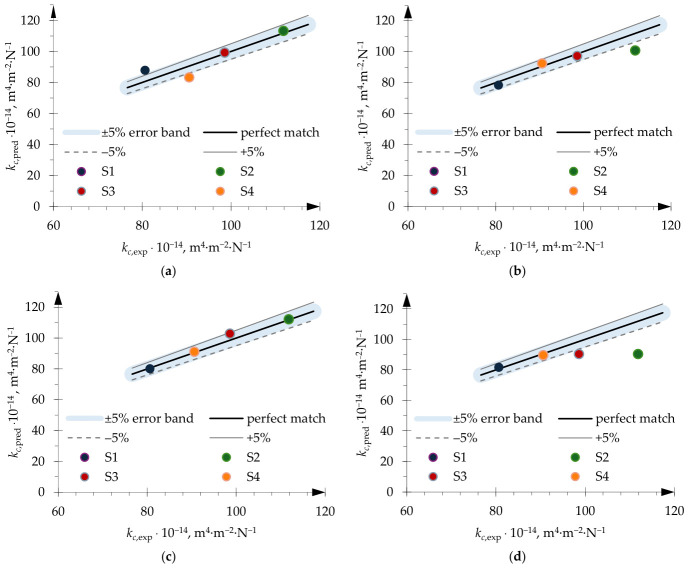
Parity plots of predicted vs. experimental values for: (**a**) method M0, (**b**) method M1, (**c**) method M2 and (**d**) method M3. The shaded area represents the ±5% error band.

A comparison of the experimental and predicted values of the wear-rate coefficient is shown in Figure 6. The results indicate that the predictions of Model M2 reproduced the experimental trend best for all specimens. Model M1 preserved the general trend of changes, but clearly underestimated the value for specimen S2. Model M0 reproduced specimens S2 and S3 well, but showed larger deviations for S1 and S4. The weakest trend reproduction was obtained for Model M3.

**Figure 7:**
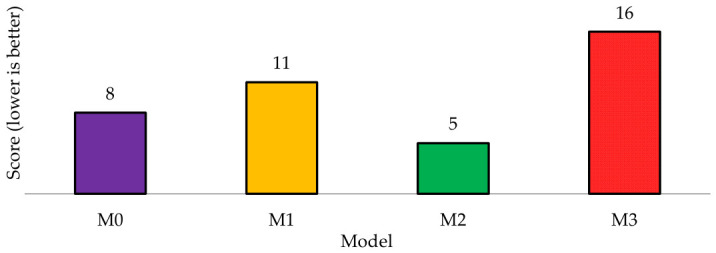
Ranking of the predictive models based on aggregated accuracy criteria. Lower score indicates better overall performance.

The aggregated model ranking is shown in Figure 7. This ranking combines information from several accuracy criteria and enables a concise assessment of the overall model quality. The lowest, and therefore best, ranking value was obtained for Model M2. Model M3 was ranked last, which is consistent with the results obtained from MAPE, RMSE and maximum error.

In summary, both the tabular and graphical analyses indicate that Model M2 provided the best agreement with the experimental results within the present dataset and calibration scheme. This improved performance may be associated with the inclusion of effective hardness and contact-related descriptors; however, because the analysed models differ simultaneously in structure, descriptors and adopted parameters, the present study does not allow the improvement to be attributed to a single modelling assumption.

Because the dataset contains only four material formulations and only two of them were used as independent validation points, the obtained prediction errors should not be interpreted as proof of general predictive capability. They demonstrate the suitability of the proposed calibration framework for ranking and comparing model structures within the investigated composition range. Therefore, Model M2 should be regarded as the best-performing formulation for the present dataset and calibration scheme, not as a universal wear-prediction law for all copper-free friction materials.

## 4. Discussion of Modelling and Validation Results

The obtained results indicate that the tribological behaviour of the analysed friction materials cannot be interpreted solely as a simple consequence of eliminating copper from the formulation. The reference specimen S1 contained 20 wt.% Cu, whereas in specimens S2–S4 copper was replaced by different Al/PTFE ratios, with the remaining constituents kept constant. This design isolated the effect of the Al/PTFE balance on the abrasive wear-rate coefficient. The highest wear was observed for specimen S2, whereas increasing the aluminium fraction and reducing the PTFE content gradually decreased the wear coefficient. Nevertheless, even the most favourable copper-free variant, S4, exhibited higher wear than the copper-containing reference material.

This trend confirms that the Al/PTFE system did not fully reproduce the tribological function of copper. Although PTFE may contribute to transfer-layer formation under favourable contact conditions, its effect depends on the mechanical stability of the near-surface composite layer, compatibility with the matrix and interaction with hard phases. The improved behaviour of S3 and S4 compared with S2 suggests that aluminium may contribute to contact stabilisation through its higher stiffness and thermal conductivity.

From a microstructural point of view, the differences in wear behaviour can be associated with the different roles of metallic, polymeric and hard constituents in the near-surface contact region. In the copper-containing reference material, copper may contribute to a more stable tribological layer and improve heat dissipation from local contact spots. In the copper-free materials, a higher PTFE fraction may support local lubrication, but excessive PTFE content can also reduce the load-bearing capacity of the near-surface composite layer and promote easier removal of the polymer-rich matrix. This mechanism can explain the higher wear rate observed for S2. In contrast, increasing the aluminium fraction in S3 and S4 may improve heat spreading and local contact stiffness, which is consistent with the gradual decrease in wear rate from S2 to S4. Because detailed post-wear microstructural observations were not performed in this study, this interpretation should be treated as a mechanism-based explanation supported by the composition–wear trends rather than as direct microstructural evidence. The modelling results confirm that wear prediction for such materials requires consideration not only of composition itself, but also of effective material and contact descriptors. Model M3, introduced as a simplified linear composition-based model, showed the weakest overall prediction performance. The high MAPE, RMSE and maximum error values indicate that a linear description of the influence of Cu, Al, PTFE and hard phases is insufficient to reproduce the nonlinear interactions occurring in the contact zone. The largest deviation of Model M3 was observed for specimen S2, for which the model strongly underestimated the wear coefficient. This means that the response of a material with high PTFE content and low aluminium content cannot be treated as a simple sum of the contributions of individual constituents.

Model M1, based on a logarithmic relationship with an Al/PTFE interaction term, provided better agreement with the experimental data than Model M3. Its structure made it possible to account for the multiplicative influence of selected constituents and the interaction between aluminium and PTFE. This allowed the general trend to be reproduced more accurately; however, the model still underestimated the wear of specimen S2. This result indicates that the Al/PTFE interaction term alone was not sufficient to fully describe the complex wear mechanisms occurring in the material with the highest PTFE content. It can therefore be assumed that the role of PTFE in the analysed composite was not exclusively film forming, but also depended on the mechanical stability of the composite surface and the presence of phases capable of carrying load.

Model M0, describing the competition between hard phases and film-forming constituents, provided a physically interpretable representation of the wear mechanism. This model reproduced the behaviour of specimens S2 and S3 relatively well and had the lowest absolute bias among the analysed variants. This means that, on average, it did not show a strong tendency either to overestimate or underestimate the experimental results. At the same time, Model M0 exceeded the ±5% error band for specimens S1 and S4. This suggests that the concept of competition between hard and film-forming phases is useful, but it does not sufficiently account for changes in contact stiffness, effective hardness and pressure distribution in the contact zone. These factors may be particularly important when comparing the copper-containing reference material with copper-free materials, in which the balance between metallic and polymeric constituents changes.

Within the analysed dataset, the best prediction accuracy was obtained for Model M2. This model achieved the lowest MAPE, RMSE and maximum error values, and all its predictions were within the adopted ±5% band. The improved performance of Model M2 may be associated with the inclusion of effective hardness and Hertzian contact descriptors. Nevertheless, because several modelling assumptions differ simultaneously between the analysed formulations, the present study does not allow a rigorous attribution of the observed improvement to any single descriptor. Consequently, the model does not describe only nominal composition, but also accounts for the effective resistance of the composite to contact loading, which is important for multi-component friction materials.

The parity plots and the analysis of percentage errors confirm this interpretation. Points corresponding to the predictions of Model M2 were within the ±5% error band for all specimens, whereas the other models showed larger deviations for selected compositions. It is particularly important that the simplified models did not produce errors uniformly across the entire dataset, but showed increased deviations for specific compositions. This means that the average error alone is not a sufficient criterion for model assessment. A model intended to support material design should provide stable predictions for individual formulations, especially when it is used to evaluate substitutes for environmentally problematic constituents such as copper.

From a practical point of view, the obtained results show that the proposed calibration procedure is useful for comparing different model structures under the same experimental conditions. The use of the same type of scaling calibration made it possible to adjust the model-response level to the experimental values while preserving the differences resulting from the adopted model structure. At the same time, the results show that calibration alone cannot compensate for an inadequate model form. Despite the use of the same calibration procedure, the prediction accuracy of the individual models differed significantly. This confirms that the quality of the physical and semi-empirical assumptions is crucial for reliable wear prediction.

It should also be noted that the adopted model parameters were selected as physically motivated model-shape descriptors rather than as globally optimised fitting constants. Consequently, the present comparison should be interpreted primarily as an assessment of alternative modelling frameworks under a common calibration strategy. Future studies should investigate parameter-identification procedures and larger validation datasets to separate the effects of model structure from those of parameter selection.

The limitations of the analysis should also be indicated. The number of investigated compositions was limited to four, and the copper-free materials differed mainly in the Al/PTFE ratio. Therefore, the developed models should be treated as calibrated predictive tools for the analysed composition range, rather than as universal wear models for all friction materials. Further validation should include a larger number of compositions, variable normal loads, different sliding distances, temperatures and counter-sample materials. It would also be advisable to link the proposed modelling approach with microstructural observations and post-wear surface analysis. This would make it possible to verify whether the predicted contributions of hard phases, film-forming constituents and effective hardness correspond to the actual wear mechanisms.

Despite these limitations, the obtained results show that Model M2 is the most reliable predictive tool for the present dataset. Its effectiveness suggests that abrasive-wear modelling of multi-component friction materials should account not only for composition, but also for effective mechanical properties and contact parameters. The proposed approach should therefore be regarded as a preliminary screening tool for copper-free candidate formulations, rather than as a final design rule.

## 5. Conclusions

The conducted study showed that replacing copper with the Al/PTFE system significantly affects the abrasive wear resistance of composite friction materials. The reference specimen S1, containing copper, exhibited the lowest wear-rate coefficient, whereas all copper-free specimens showed higher wear. Among the copper-free compositions, the highest wear rate was obtained for the specimen with the highest PTFE content and the lowest aluminium content. Increasing the aluminium fraction while decreasing the PTFE fraction led to a gradual reduction in the wear coefficient, indicating that the proportion of these constituents is crucial for shaping the tribological properties of copper-free materials.

The developed and compared predictive models showed different abilities to reproduce the experimental results. The simplest linear model, M3, was not sufficient to describe the analysed material system correctly, especially for the composition with a high PTFE content. Models M0 and M1 reproduced the general trend of changes more accurately, but still showed significant deviations for selected specimens. These results confirm that the wear of multi-component friction materials cannot be reliably predicted solely from simple composition-based relationships.

The best agreement with the experimental data was obtained for Model M2, which combines the Archard approach with effective hardness and Hertzian contact descriptors. This model achieved the lowest MAPE, RMSE and maximum error values, and all its predictions were within the ±5% error band. This means that including effective mechanical properties and contact conditions improves the model’s ability to reproduce wear changes resulting from modifications in material composition.

The proposed modelling and calibration procedure may be a useful tool for supporting the preliminary screening of new friction-material compositions. Within the analysed composition range and for the calibration scheme based on specimens S1 and S4, Model M2 provided the best compromise between accuracy, prediction stability and physical interpretability. However, due to the limited number of tested formulations and validation points, it should be treated as a calibrated screening model rather than a universal predictive law. Further validation should include a broader range of material compositions and testing conditions, independent validation datasets, post-wear microstructural analysis, and additional studies of particle emissions and environmental aspects of PTFE-containing systems.

It should be noted that the present study focused exclusively on abrasive wear behaviour and wear-rate prediction. The coefficient of friction, which is another key performance parameter of brake friction materials, was not measured within the adopted ball-cratering test procedure. Therefore, the conclusions of this work should be interpreted in terms of wear resistance rather than overall braking performance. Future studies should combine wear evaluation with friction-coefficient measurements performed under conditions more representative of real braking operation.

## Figures and Tables

**Table 1:** Compositions of prepared samples.

Component Name	S1, wt%	S2, wt%	S3, wt%	S4, wt%
Copper (Cu)	20	0	0	0
Aluminium powder (Al)	0	4	10	16
Polytetrafluoroethylene (PTFE)	0	16	10	4
Steel (0.18% C, 0.5% Si, 1.65% Mn, 0.05% P, 0.02% S, 0.08% Mo)	11	11	11	11
Aramid	18	18	18	18
Resin	19	19	19	19
Fly ash	23	23	23	23
Cast iron EN-GJS-400-12	9	9	9	9

**Table 2:** Orthogonal table for preliminary tests.

Test No.	*L* [N]	*S* [m]	*n* [rpm]
1	2	50	38
2	2	100	80
3	2	150	150
4	4	50	80
5	4	100	150
6	4	150	38
7	6	50	38
8	6	100	150
9	6	150	80

**Table 3:** Preliminary test results for S1.

Test No.	Average Crater Diameter at Repeat No., mm				
	1	2	3	4	5
1	0.39	0.37	0.40	0.41	0.38
2	0.53	0.56	0.58	0.53	0.58
3	1.06	1.09	1.18	1.18	1.15
4	0.91	1.26	1.19	1.17	1.22
5	1.41	1.45	1.31	1.37	1.31
6	1.28	1.11	1.35	1.45	1.33
7	0.86	1.28	1.14	1.38	1.06
8	0.85	0.86	0.84	0.71	0.82
9	1.93	1.85	1.88	1.86	1.91

**Table 4:** Calculated wear coefficient values.

Group Number	Sample Number	*k_c_* [10^−14^ m^4^·m^−2^·N^−1^]					Average	Standard Deviation
		Run No 1	Run No 2	Run No 3	Run No 4	Run No 5		
S1	1	81.58	79.24	76.55	78.88	81.71	80.655	±2.224
	2	80.64	83.49	79.12	79.97	83.39		
	3	82.47	81.95	79.89	83.74	77.21		
S2	1	110.33	109.57	115.72	112.68	110.75	111.811	±2.790
	2	108.67	114.38	109.94	111.35	112.93		
	3	112.96	116.39	107.41	108.77	115.31		
S3	1	104.91	97.78	96.71	97.59	99.24	98.586	±2.098
	2	98.74	96.23	100.31	98.54	97.35		
	3	99.36	97.32	96.78	99.28	98.65		
S4	1	87.39	87.67	92.76	90.81	91.44	90.579	±2.179
	2	89.66	93.47	91.55	88.62	90.68		
	3	91.24	92.11	94.39	87.48	89.41		

**Table 5:** Calculated sample volume loss per unit of friction path.

Sample Group No	*V*,·10^−12^ m^3^·m^−1^
S1	1.6131
S2	2.2362
S3	1.9717
S4	1.8116

**Table 6:** General characteristics of the predictive models.

Model	Short Name	Main Assumption	Role in the Study
M0	Hard phase–tribofilm model	Competition between abrasive hard phases and film-forming constituents.	Physically interpretable semi-empirical model.
M1	Al/PTFE interaction model	Multiplicative effect of Al, PTFE and their interaction.	Empirical model focused on copper replacement.
M2	Modified Hertz–Archard model	Wear depends on composition, effective hardness and contact pressure.	Main physically based predictive model.
M3	Linear composition model	Wear changes linearly with constituent fractions.	Simplified reference model.

**Table 7:** Prediction accuracy metrics calculated for the four predictive wear models.

Model	MAPE	RMSE *k_c,_*_pred_	Bias *k_c,_*_pred_	Max. abs.	Pred.	Rank	Comment
	[%]	[10^−14^ m^4^·m^−2^·N^−1^]		Error [%]	Within ±5%		
M0	4.7%	5.167	0.543	8.9%	2	2	
M1	4.0%	5.723	−3.175	9.8%	3	3	
M2	1.5%	2.181	1.127	4.3%	4	1	Best overall performance
M3	7.4%	11.496	−7.375	19.2%	2	4	Lowest overall performance

**Table 8:** Sample-wise signed percentage prediction errors for the analysed models.

Sample	*k_c,_*_exp_ [10^−14^ m^4^·m^−2^·N^−1^]	M0 [%]	M1 [%]	M2 [%]	M3 [%]	Best Model	Worst Model
S1	80.655	8.9%	−2.8%	−0.8%	1.3%	M2	M0
S2	111.811	1.4%	−9.8%	0.3%	−19.2%	M2	M3
S3	98.586	0.8%	−1.3%	4.3%	−8.3%	M0	M3
S4	90.579	−8.0%	2.0%	0.6%	−1.0%	M2	M0

**Table 9:** Summary of model performance in terms of the number of predictions within the ±5% error band.

Model	Predictions Within ±5%	Share Within ±5%	Samples Outside ±5%
M0	2	50%	S1, S4
M1	3	75%	S2
M2	4	100%	None
M3	2	50%	S2, S3

**Table 10:** Ranking of the predictive models according to selected criteria. Lower score indicates better overall performance.

Criterion	Best Model	Value	Second-Best Model	Value
Lowest MAPE	M2	1.5%	M1	4.0%
Lowest RMSE	M2	2.181	M0	5.167
Lowest (Bias)	M0	0.543	M2	1.127
Lowest max. error	M2	4.3%	M0	8.9%

## Data Availability

The original contributions presented in this study are included in the article. Further inquiries can be directed to the corresponding author.

## Appendix A. Mathematical Formulation of the Predictive Models

This appendix contains the detailed mathematical formulation of the models used in the main text. The material was moved from the main body of the manuscript in order to improve readability while preserving reproducibility of the modelling procedure.

### Appendix A.1. Material Input Data Used in the Calculations

The density, hardness and elastic-property values adopted for the model calculations are summarised in Table A1. These values were used as literature-based engineering input data for the mass-to-volume conversion, effective-property estimation and Hertzian contact calculations. They should be interpreted as modelling parameters rather than as directly measured properties of the produced composite specimens.

Table A1 therefore provides the complete set of material input parameters required by the models: density, hardness, Young’s modulus, Poisson’s ratio and the corresponding source or modelling assumption for each constituent. These values were used only as engineering input data for the semi-empirical calculations.

**Table A1:** Material input properties adopted for the predictive modelling calculations.

Constituent	Density [g/cm^3^]	Hardness H [GPa]	Young’s Modulus E [GPa]	Poisson Ratio ν [-]	Use/Assumption
Copper	8.96	0.80	110	0.34	Film-forming metallic constituent and heat conductor
Aluminium powder	2.70	0.25	70	0.33	Thermally conductive metallic replacement constituent
PTFE	2.20	0.05	0.5	0.46	Low-shear film-forming polymeric constituent
Steel particles	7.85	2.0	210	0.30	Hard/abrasive phase
Aramid fibres	1.44	0.20	70	0.35	Structural reinforcement; effective literature value
Resin	1.20	0.20	3.0	0.35	Polymeric matrix
Fly ash	2.30	5.0	70	0.25	Mineral hard filler
Cast iron EN-GJS-400-12	7.10	1.6	170	0.28	Hard/abrasive phase
Steel counter-sample ball	—	—	210	0.30	Used only in Hertzian contact calculation

### Appendix A.2. General Modelling Assumptions

In order to quantitatively describe the effect of the friction-material composition on abrasive wear resistance, four variants of a predictive model were developed. The output variable of the model was the abrasive wear-rate coefficient *k_c_*, determined from the ball-cratering test results. In the subsequent analysis, the volumetric material loss was assumed to be described by Archard’s relationship (Equation (A1)).

The starting point for the calculations was the conversion of the mass fractions of individual constituents into volume fractions. This step is important because the mass fraction does not directly represent the actual volume occupied by a given component in the composite structure.

The volume fraction of component *v_i_* was calculated as:

(A1) $$v_{i}=\frac{w_{i}/\rho_{i}}{\sum_{j}w_{j}/\rho_{j}}$$

where *w_i_* is the mass fraction of component *i* and *ρ_i_* is its density.

The volume fractions used in the models were nominal volume fractions calculated from the weighed mass fractions and constituent densities. They were not obtained by quantitative microstructural image analysis. Therefore, any microstructural image, if provided, should be interpreted only as qualitative support for the material description, not as a basis for determining the volume fractions.

On this basis, the constituents were divided into three functional groups used in the predictive models: hard/abrasive phases, film-forming phases and matrix-related constituents. Steel and cast iron were classified as hard metallic constituents, whereas fly ash was included in this group because it contains mineral oxide particles, mainly silica- and alumina-based phases, which may contribute to the abrasive and load-bearing response of brake-friction composites. The use of fly ash as a filler or friction-modifying constituent in brake lining materials has also been reported in previous studies [16,51,52].

Copper and PTFE were classified as film-forming constituents. Copper can stabilise the tribological contact and participate in tribolayer formation [7]. PTFE was assigned to this group because PTFE-based materials are known to transfer polymer fragments to the counter surface during sliding, forming a transfer film that may reduce adhesion and friction [53,54]. However, PTFE was treated in the model as a potential film-forming constituent, since the formation, continuity and durability of such a film depend on the contact conditions, counter-surface morphology and surrounding composite structure [54]. Resin and aramid fibres were treated as matrix-related constituents and structural reinforcement.

The effective hardness of the composite was determined from the volume fractions and hardness values of the individual constituents. Three mixing schemes were considered: linear, harmonic and power-law. The linear rule was described by:

(A2) $$H_{ef\mathrm{eff}}=\sum_{i}v_{i}H_{i}$$

the harmonic rule by:

(A3) $$H_{\mathrm{eff}}={\left(\sum_{i}\frac{v_{i}}{H_{i}}\right)}^{-1}$$

and the power-law rule by:

(A4) $$H_{\mathrm{eff}}={\left(\sum_{i}v_{i}H_{i}^{\eta }\right)}^{1/\eta }$$

where *H_i_* denotes the hardness of component *i*, *H_eff_* is the effective hardness of the composite and *η* is the mixing exponent, which controls the relative influence of softer and harder phases in the power-law mixture rule. Positive values of *η* give greater weight to harder constituents, whereas lower values make the effective hardness more sensitive to softer phases. In the final calculations, the harmonic rule was selected because it provides a more conservative estimate of effective hardness in highly heterogeneous composites containing both hard abrasive constituents and softer polymeric or film-forming phases. In such materials, the near-surface wear response may be governed by the weaker and more compliant phases; therefore, the harmonic rule better reflects the limiting effect of softer constituents on the effective load-bearing response of the friction material than a simple linear mixture rule. It should also be emphasised that *H_eff_* is a model descriptor calculated from constituent properties and mixture rules, not a directly measured hardness of the whole composite. Such an equivalent-property approach is commonly used to represent the abrasive response of multiphase materials, where the contributions of individual phases are combined to approximate the effective resistance of the material to deformation and micro-cutting [55].

### Appendix A.3. Mathematical Description of Predictive Models

This section presents four semi-empirical predictive formulations used to estimate the abrasive wear-rate coefficient *k*_c_. The models differ in the way they represent the physical and compositional factors affecting wear. In particular, they differ in whether the material response is described only by the fractions of selected constituents, by the interaction between aluminium and PTFE, or additionally by effective mechanical properties and Hertzian contact parameters.

Model M0 describes wear as a result of competition between two groups of constituents: hard phases, which may intensify abrasive wear, and film-forming phases, which may reduce adhesion and support the formation of a transfer layer. Model M1 is a logarithmic composition-based model introduced to describe the non-additive interaction between aluminium and PTFE after copper removal. Model M2 is a modified Hertz–Archard model in which the wear response depends not only on composition, but also on effective hardness and maximum Hertzian contact pressure. Model M3 is a simplified linear composition-based model used as a reference case to check whether a direct linear combination of constituent fractions is sufficient to reproduce the experimental trend.

In each model, the first calculated output is the uncalibrated wear-rate coefficient *k*_c,raw_. The term “uncalibrated” means that this value reflects the relative effect of composition, phase fractions and material descriptors predicted by a given model, but it has not yet been adjusted to the absolute experimental level. Therefore, *k*_c,raw_ should be interpreted as the model response before scaling. The transformation of *k*_c,raw_ into the final predicted value *k*_c,pred_ is performed using the calibration procedure described in Appendix A.4.

The term “model formulations” refers to four alternative semi-empirical equations developed for the analysed friction-material system. They are not intended to replace classical wear theories. Instead, they combine established tribological concepts, such as Archard-type wear and Hertzian contact, with composition-based descriptors specific to the investigated copper-containing and copper-free composite friction materials. Accordingly, the equations in Appendix A are calibrated semi-empirical formulations intended for comparative screening within the present experimental domain. They should not be used as universal wear laws unless the assumed wear mode, material system and range of operating parameters are independently validated.

#### Appendix A.3.1. Model M0—Hard-Phase and Tribological-Film Model

The first model variant, denoted as M0, is an approach based on the competition between hard phases and film-forming constituents. It was assumed that the wear rate depends, on the one hand, on the presence of hard constituents that can increase the contribution of the abrasive mechanism and, on the other hand, on the presence of constituents capable of forming a transfer layer or tribological film.

In this model, the effective fraction of the tribological film was introduced as:

(A5) $$C_{F}=\frac{f_{F}}{f_{F}+\Gamma f_{H}+\delta f_{M}+\varepsilon }$$

where *f_F_* denotes the fraction of film-forming phases, *f_H_* is the fraction of hard phases, *f_M_* is the matrix fraction, Γ defines the intensity of film degradation by hard phases, *δ* describes the contribution of the matrix to limiting the real film area and *ε* is a small numerical stabilisation constant.

The raw, uncalibrated value of the wear coefficient was determined as the sum of the baseline response, the response associated with the tribological film and the additional contribution of hard phases:

(A6) $$k_{c,\mathrm{raw}}^{\left(M0\right)}=(1-C_{F})k_{c,m}+C_{F}k_{c,F}+A_{H}f_{H}{\left(\frac{H_{\mathrm{eff}}}{H_{m}}\right)}^{\alpha_{H}}$$

where *k_c,m_* denotes the baseline wear coefficient associated with the matrix, *k_c,F_* corresponds to the regime dominated by the tribological film, *A_H_* is the coefficient describing the intensity of the hard-phase effect, *H_m_* is the matrix hardness and *α_H_* is the exponent defining the influence of the effective composite hardness.

Model M0 has a semi-empirical character. Its advantage is physical interpretability because it separately accounts for the action of phases that increase wear and phases that can reduce adhesion and friction by forming a transfer layer.

In this context, the term tribological film denotes a potential transfer layer or third-body layer formed in the contact zone from wear debris and film-forming constituents; it should not be interpreted as a separately deposited coating or as direct evidence that a continuous and stable film was formed in every test. PTFE was therefore treated as a potential transfer-film-forming constituent, because PTFE-based materials can transfer polymer fragments to the counter surface during sliding, but the formation, continuity and durability of such a layer depend on the load, counter-surface morphology, sliding conditions and the surrounding composite structure [53,54].

#### Appendix A.3.2. Model M1—Logarithmic Model with Al/PTFE Interaction

The second variant, denoted as M1, was based on a logarithmic relationship. This form was selected because the effect of replacing copper with the aluminium–PTFE system was expected to be non-additive. In multi-component friction materials, the contribution of a given constituent to wear may depend on the presence and amount of other constituents, especially when one phase promotes transfer-layer formation and another affects heat conduction and contact stability. A logarithmic formulation makes it possible to describe such multiplicative effects in an additive form after logarithmic transformation. It also reduces the risk of obtaining non-physical negative wear-rate values, because the final wear coefficient is calculated in exponential form. Therefore, the model was written in logarithmic space:

(A7) $$\ln\left(k_{c,\mathrm{raw}}^{\left(M1\right)}\right)=\ln\left(k_{c,m}\right)+a_{\mathrm{Cu}}x_{\mathrm{Cu}}+a_{\mathrm{Al}}x_{\mathrm{Al}}+a_{\mathrm{PTFE}}x_{\mathrm{PTFE}}+a_{\text{Al/PTFE}}x_{\mathrm{Al}}x_{\mathrm{PTFE}}+a_{H}f_{H}$$

After transformation, the following form was obtained:

(A8) $$k_{c,\mathrm{raw}}^{\left(M1\right)}=k_{c,m}\exp\left(a_{\mathrm{Cu}}x_{\mathrm{Cu}}+a_{\mathrm{Al}}x_{\mathrm{Al}}+a_{\mathrm{PTFE}}x_{\mathrm{PTFE}}+a_{\text{Al/PTFE}}x_{\mathrm{Al}}x_{\mathrm{PTFE}}+a_{H}f_{H}\right)$$

where *x*_Cu_, *x*_Al_ and *x*_PTFE_ denote the mass fractions of copper, aluminium and PTFE, respectively, expressed as fractions, whereas *a*_Cu_, *a*_Al_, *a*_PTFE_, *a*_Al/PTFE_ and *a_H_* are model coefficients.

The term *x*_Al_*x*_PTFE_ describes the interaction between aluminium and PTFE. This is particularly important in the analysed case because the copper-free materials S2–S4 differ mainly in the mutual proportion of these two constituents. Model M1 is empirical, but it is well suited to describing a system in which selected constituents are varied while the remaining part of the formulation is kept constant. Thus, the logarithmic form was used as a modelling choice for a non-additive and potentially multiplicative Al/PTFE effect rather than as a purely statistical transformation. The exponential form of the final equation also prevents non-physical negative values of the wear-rate coefficient.

#### Appendix A.3.3. Model M2—Modified Archard Model with a Hertzian Term

The third variant, denoted as M2, was based on Archard’s law [34] and extended by including the influence of the effective composite hardness and the maximum contact pressure determined from Hertz theory [40,41]. This model assumes that the wear rate depends not only on the material composition, but also on the relationship between the local contact pressure and the effective resistance of the composite to deformation and micro-cutting.

The material input data used to estimate the effective elastic properties of the composite and the Hertzian contact descriptors, including the Young’s modulus and Poisson’s ratio of the individual constituents, are listed in Table A1. In the first step, the effective Young’s modulus and effective Poisson’s ratio of the composite were calculated using volume-fraction-based mixture rules:

(A9) $$E_{\mathrm{eff}}=\sum_{i}v_{i}E_{i}$$

(A10) $$\nu_{\mathrm{eff}}=\sum_{i}v_{i}\nu_{i}$$

where *E*_eff_ and *ν*_eff_ denote the effective Young’s modulus and effective Poisson’s ratio of the composite, respectively. The symbols $E_{i}$ and $\nu_{i}$ refer to the Young’s modulus and Poisson’s ratio of component *i*, whereas *ν*_i_ is its volume fraction.

The reduced elastic modulus for the ball–specimen contact was then calculated as:

(A11) $$\frac{1}{E^{*}}=\frac{1-\nu_{\mathrm{eff}}^{2}}{E_{\mathrm{eff}}}+\frac{1-\nu_{b}^{2}}{E_{b}}$$

where $E^{*}$ is the reduced elastic modulus of the ball–specimen contact. The symbols *E*_b_ and *ν_b_* denote the Young’s modulus and Poisson’s ratio of the ball material, respectively.

The Hertzian contact radius was calculated from:

(A12) $$a={\left(\frac{3LR_{b}}{4E^{*}}\right)}^{1/3}$$

where *R_b_* denotes the ball radius.

The maximum contact pressure was determined as:

(A13) $$p_{0}=\frac{3L}{2\pi a^{2}}$$

The raw value of the wear-rate coefficient in Model M2 was expressed as:

(A14) $$k_{c,\mathrm{raw}}^{\left(M2\right)}=k_{c,m}{\left(\frac{H_{m}}{H_{\mathrm{eff}}}\right)}^{\alpha_{H}}\left(1+A_{H}f_{H}\right)\left(1-A_{F}f_{F}\right){\left(1+\frac{p_{0}}{H_{\mathrm{eff}}}\right)}^{m_{p}}$$

where *A_H_* describes the influence of hard phases, *A_F_* defines the effect of film-forming phases and *m_p_* is an exponent describing the effect of the relative contact-load level. Model M2 has the most physical character among the analysed variants because it combines the classical Archard law with elastic contact description and effective material properties of the composite. The adequacy of Model M2 is therefore limited to the recognised wear mode and to the investigated range of composition and normal load. It should be treated as the most appropriate calibrated model for the present dataset, not as a general predictive law for all copper-free friction materials or for other wear regimes without independent validation.

#### Appendix A.3.4. Model M3—Linear Composition-Based Model

The fourth variant, denoted as M3, was simplified and introduced as a reference model. In this case, it was assumed that the influence of constituents on the wear rate can be described by a linear combination of their fractions and an additional interaction term for the Al/PTFE system:

(A15) $$k_{c,\mathrm{raw}}^{\left(M3\right)}=k_{c,m}\left[1+b_{\mathrm{Cu}}x_{\mathrm{Cu}}+b_{\mathrm{Al}}x_{\mathrm{Al}}+b_{\mathrm{PTFE}}x_{\mathrm{PTFE}}+b_{\text{Al/PTFE}}x_{\mathrm{Al}}x_{\mathrm{PTFE}}+b_{H}f_{H}-b_{F}f_{F}\right]$$

where *b_Cu_*, *b*_Al_, *b*_PTFE_, *b*_Al/PTFE_, *b*_H_ and *b*_F_ are coefficients describing the influence of individual constituents and phase groups. The positive sign of the *b*_H_*f*_H_ term corresponds to an increase in the contribution of the abrasive mechanism with increasing hard-phase fraction. The negative sign of the *b_F_f_F_* term reflects the possible reduction in wear by film-forming phases.

Model M3 has the smallest number of physical assumptions and can be treated as a comparative model. Its advantage is simplicity, whereas its limitation is the lower ability to describe nonlinear effects, such as a change in wear regime or strong interactions between constituents.

### Appendix A.4. Model Calibration Procedure

Each of the four models first determined a raw value denoted as *kc_,_*_raw_. This value described the relative influence of the material composition and assumed phase properties, but it could differ in numerical level from the experimental values. For this reason, scaling calibration was introduced by determining the coefficient *C*:

(A16) $$k_{c,\mathrm{pred}}=Ck_{c,\mathrm{raw}}$$

where *kc*_,pred_ denotes the predicted value after calibration. In the final calculations, the coefficient C was determined using specimens S1 and S4 as calibration points. Thus, *u_k_* = 1 was assigned to S1 and S4, whereas *u_k_* = 0 was assigned to S2 and S3 during the determination of C. Specimens S2 and S3 were subsequently used to assess the ability of the calibrated models to reproduce the intermediate Al/PTFE compositions. The calibration procedure was formulated as the minimisation of the sum of squared deviations between experimental and calculated values:

(A17) $$SSE(C)=\sum_{k}u_{k}q_{k}{\left(Ck_{c,\mathrm{raw},k}-k_{c,\exp,k}\right)}^{2}$$

where *k_c_*_,exp*,k*_ is the experimental value of the wear-rate coefficient for specimen *k*, *u_k_* is the parameter selecting the specimen for calibration and *q_k_* is the weight assigned to a given specimen. The mean *_kc_* values calculated for each specimen group were used as experimental reference values. In the final comparison, equal weights were used (*q_k_* = 1) because the coefficients of variation obtained in the ball-cratering tests were low and comparable between the investigated material groups. In a more general weighted variant, the following can be assumed:

(A18) $$q_{k}=\frac{1}{s_{k}^{2}}$$

where *s_k_* denotes the standard deviation of the experimental results for specimen *k*. This approach gives greater importance in calibration to specimens with lower scatter in the experimental results.

The coefficient *C* was determined analytically from the condition of the minimum of the objective function. The following relationship was obtained:

(A19) $$C=\frac{\sum_{k}u_{k}q_{k}k_{c,\mathrm{raw},k}k_{c,\exp,k}}{\sum_{k}u_{k}q_{k}k_{c,\mathrm{raw},k}^{2}}$$

After determining the coefficient *C*, the same scaled model was applied to all specimens, both calibration and validation ones. This made it possible to assess whether a given model merely fitted the selected points or was actually able to predict the effect of compositional change on the wear rate.

### Appendix A.5. Model Parameters Adopted in the Calculations

In the developed models, the structural parameters were assumed to define the relative sensitivity of the model to the fractions of particular groups of constituents, whereas adjustment of the predicted-value level to the experimental results was performed using the calibration coefficient *C*. This means that the parameters *A_H_*, *A_F_*, Γ, *δ*, *α_H_* and *m_p_* were not independently fitted to all measurement points, but were adopted on the basis of their physical interpretation (Table A2). This approach reduces the risk of overfitting the model to a small number of specimens and preserves the comparability of the analysed variants. In addition, the effective hardness was assumed to be determined using the harmonic rule (Equation (A4)).

**Table A2:** Structural parameters used in predictive models.

Parameter	Value	Model	Meaning/Justification
*A_H_*	1.2	M0, M2	Effect of hard phases, i.e., steel, fly ash and cast iron, on intensifying the abrasive mechanism. A value greater than one indicates an increase in the contribution of abrasive wear without assuming a dominant, unlimited effect of these phases.
*A_F_*	0.8	M2, M3	Effectiveness of film-forming phases, mainly copper and PTFE, in reducing wear. A value lower than one prevents non-physical prediction of zero or negative wear.
Γ	1.0	M0	Degree of tribological-film degradation by hard phases. A neutral value means that hard phases may locally disturb the film but do not completely destroy it.
*δ*	0.5	M0	Contribution of the matrix to limiting the effective area of the tribological film. A weaker effect of the matrix than that of hard and abrasive phases was assumed.
α_H_	0.7	M0, M2	Exponent describing the influence of effective composite hardness. A value lower than one represents a sublinear effect of hardness on the wear rate.
*m_p_*	0.15	M2	Exponent of the Hertzian contact term. A weak but non-zero effect of the ratio of contact pressure to effective material hardness was assumed.
*ε*	10^−12^	M0–M3	Numerical stabilisation constant preventing division by zero. Its value does not affect the results for real material compositions.

In Model M0, an effective wear-rate coefficient for the regime dominated by the tribological film was additionally assumed, *k_c,F_* = 25 · 10^−14^ m^2^·N^−1^. This value does not describe the wear of a single constituent, but rather the effective wear level of a contact in which the transfer layer or film-forming phase plays an important role. It was adopted as a value lower than the experimental values obtained for specimens S1–S4 because a stable tribological film should reduce the intensity of wear compared with a regime dominated by abrasion.

For the resin, an approximate baseline value of *k_c,m_* = 5.14 · 10^−14^ m^2^·N^−1^ was adopted. This value was treated as a baseline matrix parameter rather than as a calibration point for the entire specimen series.

In Models M1 and M3, additional coefficients (Table A3) were used to describe the effects of copper, aluminium, PTFE, Al/PTFE interaction and the fractions of hard or film-forming phases.

**Table A3:** Empirical coefficients for models M1 and M3.

Model	Parameter	Value	Meaning
M1	*a*_Cu_	0.15	Effect of copper fraction
M1	*a*_Al_	0.85	Effect of aluminium fraction
M1	*a*_PTFE_	1.60	Effect of PTFE fraction
M1	*a*_Al/PTFE_	2.20	Interaction of the Al/PTFE system
M1	*a*_H_	0.60	Effect of hard phases
M3	*b*_Cu_	0.25	Effect of copper fraction
M3	*b*_Al_	0.90	Effect of aluminium fraction
M3	*b*_PTFE_	1.40	Effect of PTFE fraction
M3	*b*_Al/PTFE_	1.80	Interaction of the Al/PTFE system
M3	*b*_H_	1.10	Effect of hard phases
M3	*b*_F_	0.40	Effect of film-forming phases

The coefficients of Models M1 and M3 were treated as model-shape parameters rather than universal material constants. Their role was to reproduce the expected tribological trend resulting from replacing copper with the Al/PTFE system. The Al/PTFE interaction term accounts for the fact that the effects of these constituents do not have to be additive: aluminium can improve thermal conductivity and contact stability, whereas PTFE promotes the formation of a layer with a lubricating character. The final adjustment of the predicted-value level to the experimental data was performed using the calibration coefficient *C*, determined separately for each model on the basis of the specimens selected for calibration.

## References

[1] Borawski A., Mieczkowski G., Szpica D. (2023). Composites in Vehicles Brake Systems—Selected Issues and Areas of Development. Materials.

[2] Blau P.J. (2001). Compositions, Functions, and Testing of Friction Brake Materials and Their Additives.

[3] Sundarkrishnaa K.L. (2015). Friction Material Composites.

[4] Eriksson M., Jacobson S. (2000). Tribological Surfaces of Organic Brake Pads. Tribol. Int..

[5] Eriksson M., Bergman F., Jacobson S. (1999). Surface Characterization of Brake Pads after Running under Silent and Squealing Conditions. Wear.

[6] Österle W., Dörfel I., Prietzel C., Rooch H., Cristol-Bulthé A.-L., Degallaix G., Desplanques Y. (2006). Third Body Formation on Brake Pads and Rotors. Tribol. Int..

[7] Österle W., Prietzel C., Kloß H., Dmitriev A.I. (2010). On the Role of Copper in Brake Friction Materials. Tribol. Int..

[8] Borawski A. (2020). Conventional and Unconventional Materials Used in the Production of Brake Pads—Review. Sci. Eng. Compos. Mater..

[9] Idris U.D., Aigbodion V.S., Abubakar I.J., Nwoye C.I. (2015). Eco-Friendly Asbestos Free Brake-Pad: Using Banana Peels. J. King Saud Univ.—Eng. Sci..

[10] Biczó R., Kalácska G., Mankovits T. (2020). Micromechanical Model and Thermal Properties of Dry-Friction Hybrid Polymer Composite Clutch Facings. Materials.

[11] Verma P.C., Menapace L., Bonfanti A., Ciudin R., Gialanella S., Straffelini G. (2015). Braking Pad-Disc System: Wear Mechanisms and Formation of Wear Fragments. Wear.

[12] Gautier di Confiengo G., Faga M.G., Gautier G. (2022). Ecological Transition in the Field of Brake Pad Manufacturing. Sustainability.

[13] Borawski A., Szpica D., Mieczkowski G. (2024). Laboratory Tests on the Possibility of Using Flax Fibers as a Plant-Origin Reinforcement Component in Composite Friction Materials for Vehicle Braking Systems. Materials.

[14] Borawski A., Szpica D., Mieczkowski G. (2025). Polytetrafluoroethylene and Aluminum Powder as an Alternative to Copper in Car Brakes Composite Friction Materials. Materials.

[15] Aranganathan N., Mahale V., Bijwe J. (2016). Effects of Aramid Fiber Concentration on the Friction and Wear Characteristics of Non-Asbestos Organic Friction Composites Using Standardized Braking Tests. Wear.

[16] Mohanty S., Chugh Y.P. (2007). Development of Fly Ash-Based Automotive Brake Lining. Tribol. Int..

[17] Kukutschová J., Roubíček V., Malachová K., Pavlíčková Z., Holuša R., Kubačková J., Mička V., MacCrimmon D., Filip P. (2009). Wear Mechanism in Automotive Brake Materials, Wear Debris and Its Potential Environmental Impact. Wear.

[18] Kukutschová J., Roubíček V., Mašláň M., Jančík D., Slovák V., Malachová K., Pavlíčková Z., Filip P. (2010). Wear Performance and Wear Debris of Semimetallic Automotive Brake Materials. Wear.

[19] Gilardi R., Alzati L., Thiam M., Brunel J.-F., Desplanques Y., Dufrénoy P., Sharma S., Bijwe J. (2012). Copper Substitution and Noise Reduction in Brake Pads: Graphite Type Selection. Materials.

[20] Wahlström J., Söderberg A., Olander L., Jansson A., Olofsson U. (2010). A Pin-on-Disc Simulation of Airborne Wear Particles from Disc Brakes. Wear.

[21] Lyu Y., Leonardi M., Wahlström J., Gialanella S., Olofsson U. (2020). Friction, Wear and Airborne Particle Emission from Cu-Free Brake Materials. Tribol. Int..

[22] Lohmann R., Cousins I.T., Dewitt J.C., Glüge J., Goldenman G., Herzke D., Lindstrom A.B., Miller M.F., Ng C.A., Patton S. (2020). Are Fluoropolymers Really of Low Concern for Human and Environmental Health and Separate from Other PFAS?. Environ. Sci. Technol..

[23] Henry B.J., Carlin J.P., Hammerschmidt J.A., Buck R.C., Buxton L.W., Fiedler H., Seed J., Hernandez O. (2018). A Critical Review of the Application of Polymer of Low Concern and Regulatory Criteria to Fluoropolymers. Integr. Environ. Assess. Manag..

[24] Aranganathan N., Bijwe J. (2016). Development of Copper-Free Eco-Friendly Brake-Friction Material Using Novel Ingredients. Wear.

[25] Mahale V., Bijwe J., Sinha S. (2019). A Step towards Replacing Copper in Brake-Pads by Using Stainless Steel Swarf. Wear.

[26] Mahale V., Bijwe J. (2020). Exploration of Plasma Treated Stainless Steel Swarf to Reduce the Wear of Copper-Free Brake-Pads. Tribol. Int..

[27] Kalel N., Bhatt B., Darpe A., Bijwe J. (2022). Argon Low-Pressure Plasma Treatment to Stainless Steel Particles to Augment the Wear Resistance of Cu-Free Brake-Pads. Tribol. Int..

[28] Kalel N., Bhatt B., Darpe A., Bijwe J. (2021). Copper-Free Brake-Pads: A Break-Through by Selection of the Right Kind of Stainless Steel Particles. Wear.

[29] Naidu M., Bhosale A., Munde Y., Salunkhe S., Hussein H.M.A. (2023). Wear and Friction Analysis of Brake Pad Material Using Natural Hemp Fibers. Polymers.

[30] Satapathy B.K., Bijwe J. (2006). Composite Friction Materials Based on Organic Fibres: Sensitivity of Friction and Wear to Operating Variables. Compos. Part A Appl. Sci. Manuf..

[31] Borawski A. (2021). Impact of Operating Time on Selected Tribological Properties of the Friction Material in the Brake Pads of Passenger Cars. Materials.

[32] Borawski A., Szpica D., Mieczkowski G. (2022). Research on Tribological Features of Brake Friction Materials—Comparison of the Results Obtained with the Pin-On-Disc and Ball-Cratering Methods. Mechanika.

[33] Borawski A. (2023). Study of the Influence of the Copper Component’s Shape on the Properties of the Friction Material Used in Brakes. Materials.

[34] Archard J.F. (1953). Contact and Rubbing of Flat Surfaces. J. Appl. Phys..

[35] Hatam A., Khalkhali A. (2018). Simulation and Sensitivity Analysis of Wear on the Automotive Brake Pad. Simul. Model. Pract. Theory.

[36] Sawczuk W., Merkisz-Guranowska A., Ulbrich D., Kowalczyk J., Rilo Cañás A.-M. (2022). Investigation and Modelling of the Weight Wear of Friction Pads of a Railway Disc Brake. Materials.

[37] Biczó R., Kalácska G., Mankovits T. (2021). Effects of Automotive Test Parameters on Dry Friction Fiber-Reinforced Clutch Facing Surface Microgeometry and Wear. Polymers.

[38] Kalácska G., Biczó R., Mankovits T. (2022). Effects of Automotive Test Parameters on Dry Friction Fiber-Reinforced Clutch Facing Surface Microgeometry and Wear—Part 3 Tribological Parameter Correlations. Polymers.

[39] Grześ P., Kuciej M. (2025). FE Analysis of the Brake Pad and Brake Disc Wear with Thermal-Structure Interaction. Wear.

[40] Hertz H. (1882). Ueber Die Berührung Fester Elastischer Körper. J. Reine Angew. Math..

[41] Johnson K.L. (1985). Contact Mechanics.

[42] AbuBakar A.R., Ouyang H. (2008). Wear Prediction of Friction Material and Brake Squeal Using the Finite Element Method. Wear.

[43] Borawski A. (2016). Suggested Research Method for Testing Selected Tribological Properties of Friction Components in Vehicle Braking Systems. Acta Mech. Autom..

[44] Osuch-Słomka E., Ruta R., Słomka Z. (2013). The Use of a Modern Method of Designing Experiments in Ball-Cratering Abrasive Wear Testing. Proc. Inst. Mech. Eng. Part J J. Eng. Tribol..

[45] Osuch-Słomka E. (2012). Abrasive Wear Testing of Antiwear Coatings by Ball-Cratering-Method. Tribologia.

[46] Trezona R.I., Allsopp D.N., Hutchings I.M. (1999). Transitions between Two-Body and Three-Body Abrasive Wear: Influence of Test Conditions in the Microscale Abrasive Wear Test. Wear.

[47] Adachi K., Hutchings I.M. (2003). Wear-Mode Mapping for Th Micro-Scale Abrasion Test. Wear.

[48] Mitra A. (2021). Fundamentals of Quality Control and Improvement: Fifth Edition.

[49] (2008). Advanced Technical Ceramics—Methods of Test for Ceramic Coatings—Part 6: Determination of the Abrasion Resistance of Coatings by a Micro-Abrasion Wear Test.

[50] Osuch-Słomka E. (2011). Proposed Method for Determining the Values of Tests for the Ball-Cratering Metod. Tribologia.

[51] Choosri S., Sombatsompop N., Wimolmala E., Thongsang S. (2019). Potential Use of Fly Ash and Bagasse Ash as Secondary Abrasives in Phenolic Composites for Eco-Friendly Brake Pads Applications. Proc. Inst. Mech. Eng. Part D J. Automob. Eng..

[52] Yilmaz A.C. (2022). Effects of Fly Ash Introduction on Friction and Wear Characteristics of Brake Pads. Int. J. Automot. Eng. Technol..

[53] Ye J., Burris D.L., Xie T. (2016). A Review of Transfer Films and Their Role in Ultra-Low-Wear Sliding of Polymers. Lubricants.

[54] Li H.L., Yin Z.W., Jiang D., Jin L.Y., Cui Y.Q. (2015). A Study of the Tribological Behavior of Transfer Films of PTFE Composites Formed under Different Loads, Speeds and Morphologies of the Counterface. Wear.

[55] Axén N., Jacobson S. (1994). A Model for the Abrasive Wear Resistance of Multiphase Materials. Wear.

